# Viral infection states, immune control, and adult cognition: evidence across latency, persistence, and post-acute responses

**DOI:** 10.3389/fimmu.2026.1936082

**Published:** 2026-09-18

**Authors:** Yikai Chen, Shaowei Li, Hao Lin, Fangcheng Zou, Wenxin Wang

**Affiliations:** 1 The Second Affiliated Hospital of Henan Medical University, Henan Key Lab of Biological Psychiatry (Henan Medical University), Xinxiang, Henan, China; 2 Center for Precision Medicine, School of Medicine and School of Biomedical Sciences, Huaqiao University, Xiamen, Fujian, China; 3 The Children’s Hospital, Zhejiang University School of Medicine, Hangzhou, Zhejiang, China; 4 Fujian Medical University, Fuzhou, Fujian, China; 5 Beijing Key Laboratory of Intelligent Drug Research and Development for Mental Disorders, National Clinical Research Center for Mental Disorders, National Center for Mental Disorders, Beijing Anding Hospital, Capital Medical University, Beijing, China; 6 Brain Institute, Henan Academy of Innovations in Medical Science, Zhengzhou, Henan, China

**Keywords:** adult cognition, antiviral therapy, immune control, reactivation, vaccination, viral infection states, viral latency, viral persistence

## Abstract

Viral infections can leave latent genomes, persistent reservoirs, or prolonged host responses after the presenting illness has resolved. This Review organizes their relevance to adult cognition around three overlapping infection-state prototypes: latency with episodic reactivation, chronic persistence, and acute infection with post-acute host responses. We examine how local antiviral surveillance, antigen-driven T-cell dysfunction, innate immune memory, and inflammatory resolution shape neurovascular and glial responses. Complement-mediated synaptic remodeling, proteostatic disruption, and immunometabolic changes provide additional mechanistic links. The synthesis distinguishes persistent cognitive symptoms, longitudinal cognitive trajectories, and clinical or biomarker-defined neurodegeneration. A dedicated vaccination section examines pathogen-specific protection, adjuvant biology, and heterologous immune effects, while antiviral studies address cognitive change during viral suppression or after clearance. These findings motivate event-triggered studies of reactivation, treatment-anchored studies of persistence, and acute-to-recovery studies of post-infectious cognition, complemented by targeted experimental perturbation. Linking infection state, immune function, and outcome-specific follow-up provides a practical route from broad epidemiological associations to testable mechanisms and modifiable biological processes.

## 1 Introduction

Dementia prevention increasingly focuses on exposures that operate before clinical diagnosis. An estimated 57.4 million people were living with dementia in 2019, with approximately 152.8 million projected by 2050 (1). Established prevention frameworks emphasize vascular, sensory, behavioral, metabolic, and social determinants (2). Viral infection adds a temporal dimension: clinical recovery can coexist with a latent genome, persistent antigen, or a prolonged immune response (3, 4). The exposure relevant to cognition may therefore recur or evolve long after the initial illness.

Population studies connect infection history with cognitive outcomes across several settings. In the Northern Manhattan Study, a serology-based infectious-burden index was associated with cognitive performance and later memory decline (5, 6). National biobank analyses linked several viral exposures to neurodegenerative diagnoses (7). These associations motivate a mechanistic question: which component of infection history—reactivation, persistent antigen, or the host response—accounts for subsequent brain and cognitive change?

The three prototypes describe overlapping infection histories. Infection state captures viral persistence and activity; immune control captures the host processes that contain or resolve them. Adult cognition includes persistent symptoms, longitudinal cognitive change, and neurodegenerative outcomes, each with its own timescale. This narrative synthesis connects adult infection histories with these outcomes and draws on selected encephalitis models to develop the underlying mechanisms.

## 2 Three viral infection-state prototypes

Infection state identifies the biological transition available for study. Herpesvirus latency can alternate with brief reactivation, treated HIV can retain reservoirs despite plasma suppression, and SARS-CoV-2 infection can be followed by persistent antigen or host abnormalities (3, 4, 8). These states direct attention to containment, recovery during suppression or after clearance, and resolution after acute infection. **Table 1** connects each transition with an intervention focus, research question, and longitudinal design.

**Table 1:** Infection states, research questions, and study designs.

Infection state	Biology and readouts	Intervention focus	Research question	Study design
Latency/reactivation	Episodic shedding; local T-cell control (9–11).	Suppress reactivation	How does local control shape event-related cognitive change?	Event-triggered, within-person follow-up
Chronic persistence	HIV reservoir/CSF activity; HCV clearance and recovery (8, 12).	Viral suppression or HCV clearance	Does immune recovery track cognitive improvement?	Treatment-anchored serial follow-up
Acute/post-acute responses	Residual antigen; host-response resolution (4, 13, 14).	Prevent infection/severity; study host recovery	How do antigen and immune trajectories relate to recovery?	Acute-to-recovery follow-up

### 2.1 Virus-specific immune control and compartmentalization

Effective antiviral control depends on antigen recognition, effector function, and tissue access. Dense human HSV-2 sampling demonstrated rapid local expansion and containment of shedding episodes, with tissue CD8 T-cell organization and cytokine responses contributing to control (9, 10, 15). Reduced functional T-cell receptor–epitope interaction was associated with impaired activation and herpes zoster in a human VZV study (11). Together, these studies identify antigen recognition and local effector function as measurable components of containment.

Antiviral effectors can also preserve the infected cell. In experimental neuronal HSV-1 latency, CD8 T-cell granzyme B cleaved the viral regulatory protein ICP4 and inhibited reactivation without inducing neuronal apoptosis (16). This noncytolytic mechanism couples viral restriction with neuronal survival. It provides a concrete experimental basis for distinguishing suppression of viral gene expression from elimination of infected cells when studying neural consequences.

Persistent antigen exposure and immune aging can impair T-cell responsiveness through different mechanisms. TOX-dependent transcriptional and epigenetic programming supports CD8 T-cell exhaustion, whereas p38 signaling was linked to impaired proliferation and autophagy in senescent human CD8 T cells (17, 18). Exhaustion and senescence therefore differ from differentiation states that retain immediate effector activity. Combining antigen-specific functional assays with phenotype or transcriptional measurements can resolve these differences when evaluating antiviral control.

Humoral and innate lymphocyte responses complement T-cell measurements. CMV-associated NK-cell populations show adaptive epigenetic diversification and altered effector responses (19). Human VZV antigen-challenge and systems-level vaccination studies likewise show that antibody and cellular measurements capture different components of immunity (20, 21). Antibody kinetics, neutralization, and NK-cell assays provide focused readouts of these complementary responses.

Anatomical compartment determines what an immune measurement represents. Mucocutaneous HSV studies characterize local containment, while HIV cohorts demonstrate that cerebrospinal-fluid (CSF) viral activity can diverge from plasma suppression (8, 9, 22). Blood, accessible tissue, CSF, and barrier imaging therefore provide complementary views of systemic immunity, local surveillance, and central involvement. Paired measurements in focused substudies can establish how an accessible marker relates to the compartment relevant to cognition.

These findings motivate state-matched measurements. Human HSV studies support event-related shedding and local-control assays; HIV studies support reservoir and CSF measurements during systemic suppression; and post-acute COVID-19 studies support repeated antigen and immune profiling (4, 8, 10, 13, 23). **Tables 1** and **2** translate these empirical precedents into focused readouts and sampling strategies.

**Table 2:** State-matched measurements and sampling.

Domain	Key measures	Sampling window	Purpose
Viral activity	PCR, antigen, shedding, clinical reactivation (4, 9).	Baseline, event, early recovery	Locate viral activity in time
Adaptive immune control	Virus-specific CD4/CD8 function; antigen-specific antibodies (10, 20).	Baseline and post-event or post-intervention	Measure functional antiviral responses
Innate responses	Interferon, myeloid activation, pathway-specific sensing assays (24).	Acute/reactivation and recovery	Track activation and resolution
Tissue and vascular responses	Barrier markers, perfusion/permeability imaging, glial or neural markers (25, 26).	Baseline and serial follow-up	Link brain-interface changes to cognition
Cognitive outcomes	Domain scores, symptoms, and disease-stage measures (14, 27).	Matched to symptoms, trajectory, or incidence	Measure recovery and progression
T-cell function and repertoire	Proliferation, cytokines, cytotoxicity, phenotype, repertoire (11, 17, 18).	Baseline, event, post-intervention	Resolve effector activity and renewal
Humoral and NK-cell responses	Antibody responses and NK-cell function (19, 20).	Baseline, acute phase, recovery	Characterize complementary immune responses
Compartmentalized immunity	Paired blood–CSF or accessible-tissue assays (8, 14, 22).	Matched visits in nested substudies	Connect systemic and tissue responses
State-matched profiles	Reactivation: shedding/control; persistence: reservoir/recovery; post-acute: antigen/resolution (13, 28).	Event-triggered, treatment-anchored, or acute-to-recovery	Link infection state to its biological transition

### 2.2 Latency and episodic reactivation

HSV-1 illustrates the distinction between lifelong exposure and intermittent activity. Epidemiological syntheses associate selected HSV-1 exposure measures with Alzheimer disease, and longitudinal cohorts have examined latent infection in relation to cognitive change (29, 30). Experimental herpesvirus studies link infection to amyloid-beta responses and altered synaptic gene regulation (31, 32). Relating documented reactivation to subsequent host and cognitive responses would connect these mechanistic findings to human infection histories.

VZV provides a clinically observable reactivation event and an intervention that reduces herpes zoster. Infection–dementia estimates differ across studies, whereas several vaccination designs report lower subsequent dementia incidence (33–36). This contrast makes VZV useful for examining whether prevention of recurrent inflammatory exposure contributes to cognitive outcomes. Section 4 develops the vaccination evidence and its candidate immune mechanisms.

CMV illustrates durable immune remodeling. CMV-specific CD4 and CD8 T cells can occupy substantial fractions of the memory compartment, and persistence is associated with age-related T-cell differentiation (37, 38). Enhanced influenza-vaccine responses reported in some settings further connect CMV history with later immune responsiveness (39). These observations support studying immune allocation and response quality in immunocompetent adults, alongside the distinct biology of CMV end-organ disease in immunocompromised hosts (40).

Latency is actively maintained through restricted viral gene expression and immune evasion (3). Experimental viral interference with antigen presentation alters tissue-resident memory formation, while neuronal hyperexcitability and interleukin-1 signaling can trigger HSV-1 reactivation (41, 42). These findings support a feedback model: local surveillance constrains reactivation, and inflammatory or neuronal stress changes the threshold for escape. Sampling around an episode can determine which process changes first.

### 2.3 Chronic viral persistence

HIV separates suppression of circulating replication from cognitive recovery. Cognitive disorders remained detectable in the CHARTER cohort during potent antiretroviral therapy, with contemporary interpretation emphasizing comorbidity and heterogeneous impairment (43–45). Reservoir and CSF-escape studies identify infection-related processes beyond plasma viral load (22, 23). The central question is which residual viral, inflammatory, or nonviral processes explain cognitive trajectories during treatment.

Treated HIV also links persistent infection to metabolic and barrier-related pathways. Epigenetic age acceleration changes after antiretroviral initiation, and kynurenine-pathway activity is associated with cognition in virally suppressed women (46, 47). Gut barrier dysfunction and chronic immune activation were associated with neuroinflammation in ART-suppressed SIV infection (48). These findings motivate paired viral, myeloid, and metabolic measurements in HIV studies, with microbial translocation and treatment effects interpreted in their clinical context.

HCV provides a contrasting transition from chronic infection to clearance after finite treatment. Cognitive changes can accompany hepatic, inflammatory, metabolic, and neuropsychiatric effects of chronic HCV (49). A prospective before-and-after study reported improved cognition and depression after successful direct-acting antiviral treatment (12). Documented clearance anchors the within-person assessment of recovery. HIV suppression and HCV clearance thus offer complementary models of the relationship between viral control and host normalization.

### 2.4 Acute infection and post-acute host responses

SARS-CoV-2 demonstrates prolonged brain-related changes after acute respiratory illness. Health-record studies documented neurological outcomes, and pre-infection and post-infection UK Biobank imaging identified longitudinal brain changes (50, 51). Experimental respiratory infection produced persistent glial and myelin abnormalities, while human studies identified altered perfusion and barrier integrity in cognitively affected individuals (25, 26, 52). Together, these findings support systemic-to-brain pathways alongside local neural responses.

Post-acute abnormalities vary in timing and biological origin. Circulating spike antigen has been detected in a subset of affected individuals, and early immune or virological features have been associated with later symptoms (4, 13, 53). Convalescent studies also identify altered T-cell dynamics, inflammatory transcription, and immune profiles (54–56). Repeated antigen and host measurements can distinguish continued antigen exposure from a response that persists after its initiating stimulus diminishes.

Cognition-specific studies connect these biological trajectories to clinical outcomes. Prospective clinical and CSF single-cell profiling associated post-COVID cognitive impairment with immune patterns distinct from an Alzheimer-type biomarker profile (14). Symptoms and performance can evolve for years after hospitalization, and decline has also been reported after mild symptomatic infection (57, 58). Recovery and persistence within a defined cognitive phenotype are therefore informative contrasts for post-acute studies.

### 2.5 Distinct cognitive outcome phenotypes

Persistent symptoms, cognitive trajectories, and neurodegeneration capture different outcomes (**Table 3**). Post-acute attention, processing-speed, or memory difficulties can fluctuate with fatigue, sleep, mood, and recovery (14, 57). Multi-year trajectories instead describe within-person cognitive change, as in infectious-burden and herpesvirus cohorts (6, 59). Symptom reversibility and a slower rate of decline consequently require different follow-up even when assessed with overlapping tests.

**Table 3:** Cognitive outcomes and longitudinal assessment.

Cognitive outcome	Measures	Follow-up	Study objective	Design focus
Acute/post-acute symptoms	Attention, memory, processing speed, and symptoms (14, 57).	Days to months or longer	Symptom burden and reversibility	Fatigue, mood, and practice effects
Cognitive trajectory	Repeated domain and global scores (6, 59).	Years	Rate of change and recovery	Repeated testing and retention
Dementia/neurodegeneration	Diagnosis, AD biomarkers, atrophy, and function (36, 60).	Years to decades	Clinical incidence and disease progression	Disease stage and competing mortality

Clinical dementia and biomarker-defined neurodegeneration add complementary disease-stage information. Alzheimer biomarkers can change long before diagnosis, whereas recorded dementia also reflects survival and clinical ascertainment (60–62). Practice effects and selective dropout influence repeated cognitive scores (27, 63). Aligning the endpoint and analysis with the intended outcome allows symptom recovery, longitudinal change, and neurodegenerative progression to be evaluated separately.

## 3 Immune mechanisms linking infection state to brain function

Different infection states can converge on immune and vascular pathways with distinct initiating signals. Treated lentiviral infection illustrates persistent myeloid and barrier-related stress, respiratory SARS-CoV-2 models show sustained glial responses, and encephalitis models establish complement-dependent synaptic loss (48, 52, 64). **Figure 1** connects infection states to immune imbalance, brain-interface signaling, glial and vascular stress, and brain and cognitive outcomes. **Table 4** identifies the corresponding evidence by infection state and pathway.

**Figure 1:**
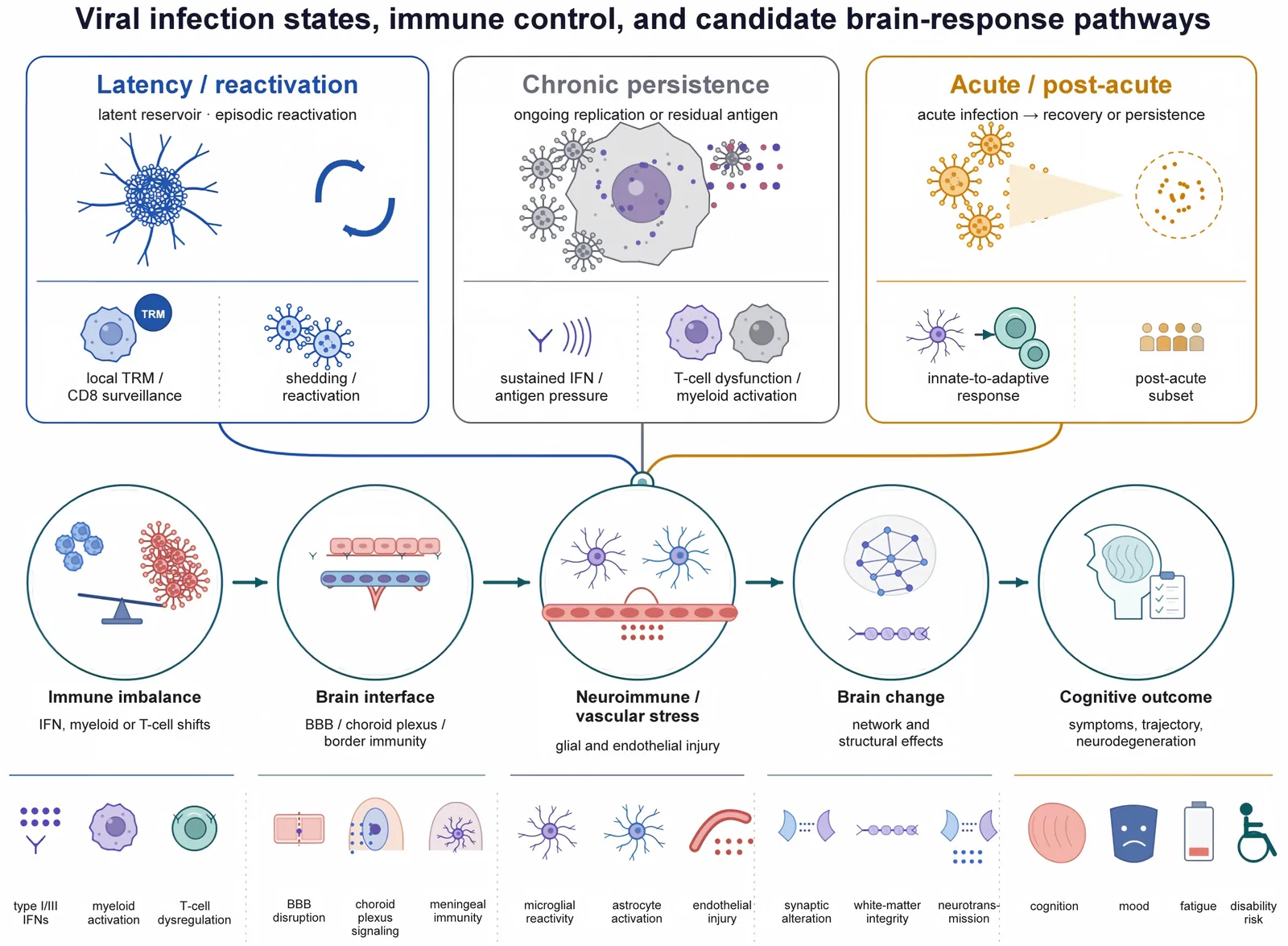
Viral infection states, immune control, and brain-response pathways. Three overlapping infection states—latency with episodic reactivation, chronic persistence, and acute infection with post-acute responses—are organized by local surveillance, antigen pressure, and immune recovery. The central schematic links immune imbalance with brain-interface signaling, glial and endothelial stress, neural changes, and cognitive outcomes. Lower illustrations summarize associated immune, vascular, synaptic, white-matter, and functional processes. BBB, blood–brain barrier; IFN, interferon; TRM, tissue-resident memory T cells.

**Table 4:** Infection-state evidence and mechanistic targets.

Pathway	Latency/reactivation	Chronic persistence	Acute/post-acute	Mechanistic focus
Immune remodeling and activation	CMV T-cell expansion (37).	Treated HIV; SIV neuroinflammation (44, 48).	Post-COVID immune and glial changes (14, 52).	Persistence and resolution
Neurovascular dysfunction	CMV-associated systemic inflammation (65).	HIV pericyte–endothelial model (66).	Post-COVID BBB/perfusion changes; lung–brain model (25, 26, 67).	Barrier integrity and perfusion
Complement-linked synaptic remodeling	—	—	C3/C3aR-dependent synapse loss in a West Nile model (64).	Synaptic tagging and engulfment
Proteostasis and immunometabolism	HSV amyloid responses; autophagy (31, 68).	Kynurenine–cognition association in HIV (47).	Kynurenine–cognition association after tick-borne encephalitis (69).	Protein clearance and metabolism

Findings are grouped by infection state and pathway. —, no included state-specific study; BBB, blood–brain barrier; C3aR, complement C3a receptor.

### 3.1 Antiviral sensing, immune memory, and inflammatory resolution

Antiviral sensing can sustain inflammation when activation persists in vulnerable tissue. In severe COVID-19 samples and a lung-on-chip model, cGAS–STING activation was linked to type I interferon production and tissue injury; endothelial sensing in the model involved mitochondrial DNA release (24). This secondary DNA-sensing mechanism explains how an RNA-virus infection can engage cGAS–STING and prolong inflammatory signaling beyond the initial antiviral response.

The consequences of sustained signaling depend on the receiving cells. Type I interferon promoted complement-associated microglial responses and synapse loss in Alzheimer models, while cGAS–STING manipulation altered inflammation and neurodegenerative phenotypes in aging mice (70, 71). These nonviral models identify experimentally tractable intermediates between prolonged innate activation and neural injury. Infection-focused studies can test whether those intermediates respond to measured viral or inflammatory activity.

Prior inflammation can reprogram responses to later challenges. Trained immunity involves durable transcriptional, epigenetic, and metabolic changes in innate responses (72, 73). In mice, systemic inflammatory stimulation induced microglial training or tolerance lasting months and altered subsequent amyloid and stroke pathology (74). Because exposure history changed the direction of the response, immune memory can amplify or restrain later injury. Response quality and recovery kinetics therefore complement the number of prior infections.

Human post-acute studies make inflammatory resolution a measurable trajectory. Longitudinal profiling identified complement dysregulation and thromboinflammatory signals in active long COVID, while cognitive cohorts identified CSF immune changes and altered barrier integrity (14, 25, 28). Tracking these abnormalities alongside antigen and cognition can show whether host recovery follows declining antigen, remains delayed, or proceeds independently of the measured antigen signal.

### 3.2 The neurovascular interface

The neurovascular interface connects peripheral inflammation with brain physiology. Endothelial cells regulate permeability and leukocyte traffic, while pericytes support capillary function and endothelial stability (75). A SARS-CoV-2 lung–brain system demonstrated barrier injury and neuroinflammation, and HIV experiments implicated pericyte infection in endothelial disruption (66, 67). These models localize cellular routes from infection to vascular dysfunction and an altered neural environment.

Human imaging provides complementary evidence for this vascular route. Dynamic contrast-enhanced MRI identified barrier disruption in long COVID-associated cognitive impairment, with patient-derived assays supporting endothelial inflammatory effects (25). Arterial-spin-labeling MRI identified hypoperfusion in another cohort, and a general-population meta-analysis associated endothelial-dysfunction markers with cognition (26, 76). Permeability, perfusion, and endothelial activation provide complementary measures for investigating vascular contributions to cognitive change.

Baseline vascular reserve may modify the cognitive impact of inflammatory exposure. Age-related barrier vulnerability and biobank associations between cardiovascular health, infection burden, and dementia support this interaction hypothesis (77, 78). Repeated barrier or perfusion measurements alongside vascular risk and cognition could test whether a comparable infection-related response produces greater functional change in people with lower reserve.

### 3.3 Complement and synaptic remodeling

Complement provides an experimentally defined route from inflammation to synaptic loss. In a West Nile encephalitis model, C3 and C3a-receptor signaling contributed to microglial uptake of presynaptic terminals and hippocampal synapse loss in animals with memory impairment (64). Synapse-targeted complement inhibition and the complement-regulating protein NPTX2 have also been studied in demyelinating and neurodegenerative models (79, 80). These perturbations distinguish synaptic tagging from microglial engulfment as testable steps.

Cytokines can alter synapses through intracellular gene regulation. In a mouse model of recurrent brain HSV-1 reactivation, IL-1-beta signaling was linked to nuclear HDAC4 accumulation and MeCP2-dependent repression of plasticity-related genes. IL-1-receptor blockade prevented molecular and synaptic abnormalities and improved selected later memory outcomes (32). This pathway connects a defined inflammatory mediator to synaptic physiology and behavior, complementing the extracellular complement-dependent mechanism.

Complement research connects questions at systemic and synaptic scales. The West Nile model resolves C3-dependent synaptic removal, while longitudinal long-COVID profiling characterizes circulating complement and vascular responses (28, 64). Paired systemic and central readouts, followed by targeted pathway perturbation, can test how these processes relate to post-infectious cognition. **Table 4** organizes the representative findings and mechanistic targets.

### 3.4 Proteostatic and immunometabolic pathways

Protein handling links antiviral defense with neuronal resilience. Autophagy and lysosomal pathways participate in antiviral defense and are themselves modified by viruses (68, 81). HSV-related experiments connect infection with amyloid-beta responses, with broader evidence describing proteostatic and neuroinflammatory interactions (31, 82, 83). Measurements of autophagic flux and protein clearance can determine how infection changes this balance over time.

Immunometabolism provides a measurable link between infection-related host responses and cognition. Kynurenine-pathway activity is associated with cognition in virally suppressed women with HIV and with persistent impairment after tick-borne encephalitis (47, 69). These associations nominate kynurenine metabolism for comparative studies combining targeted metabolites with tissue-specific sampling. Paired systemic and CSF measurements could test whether metabolic recovery accompanies inflammatory resolution and cognitive improvement.

## 4 Exposure history, disease stage, and temporal direction

Exposure history contextualizes current viral activity. Community studies report pathogen-specific and cumulative cognitive associations, including differing HSV-1 and CMV findings (59, 84, 85). Broad antibody profiling such as VirScan characterizes exposure breadth, whereas targeted virological measurements characterize current activity (86, 87). Combining these layers distinguishes lifelong immune experience from the episode being related to cognition.

Disease stage changes the possible direction of an association. Alzheimer biomarkers precede symptoms, and neuroinflammation and barrier abnormalities are part of neurodegenerative biology (60, 75, 88). In the Dominantly Inherited Alzheimer Network, baseline HSV-1 seropositivity was not associated with faster cognitive decline or whole-brain atrophy (89). Longitudinal activity measurements can therefore test whether infection-related signals precede brain changes, follow them, or modify established disease.

Exposure sequence may also shape later responses. Experimental coinfection altered CD8 T-cell response variability and disease outcomes, while herpesvirus research describes interactions with latency–reactivation dynamics (90, 91). These observations motivate selected analyses of exposure order and interval alongside the principal within-person comparison. **Figure 2** organizes the observable transitions around reactivation, chronic persistence, and acute recovery, together with intervention and study-design options.

**Figure 2:**
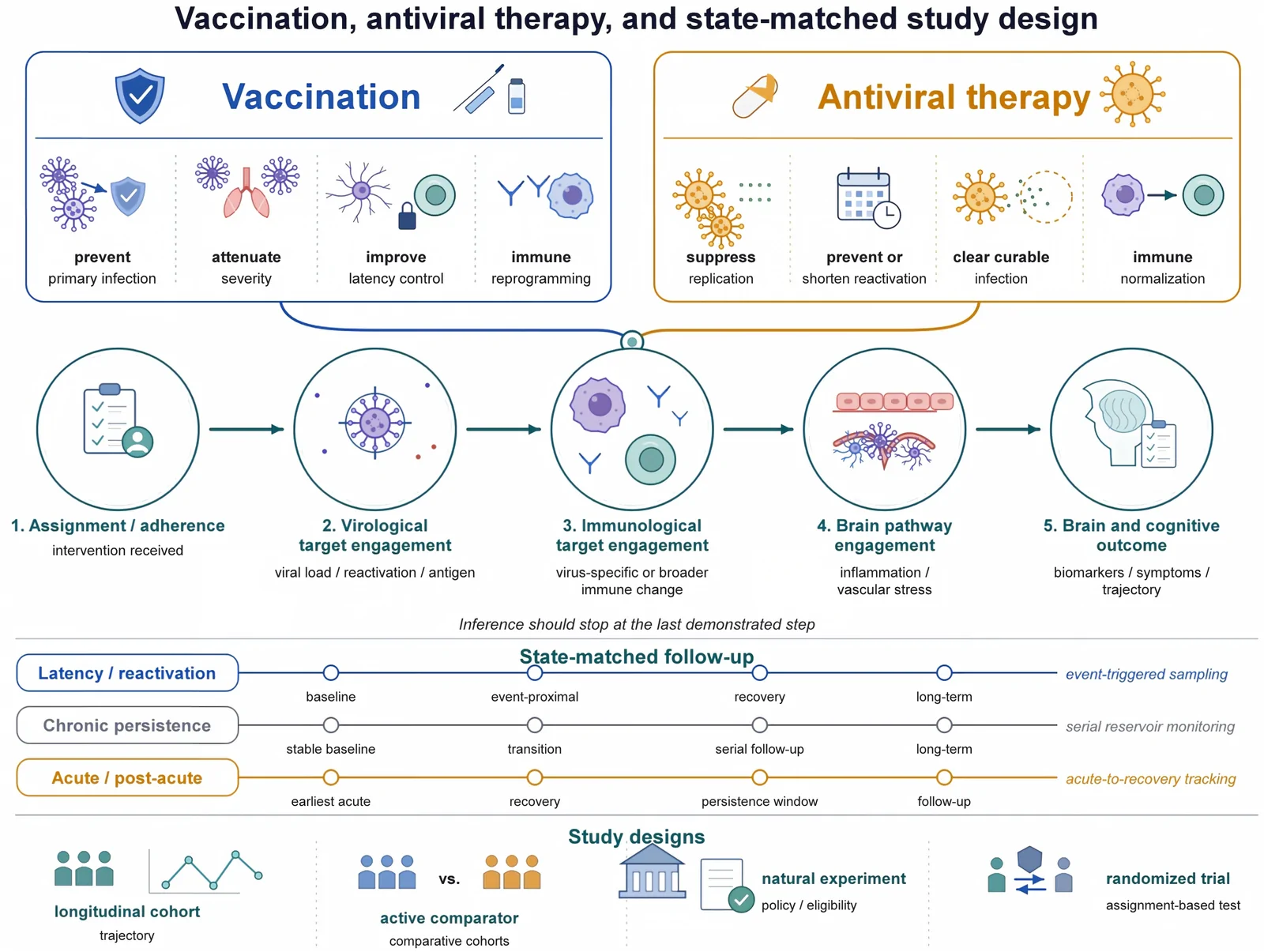
Vaccination, antiviral therapy, and state-matched study design. The upper panels summarize vaccination and antiviral actions on infection, reactivation, disease severity, and immune responses. Five assessment stages connect assignment and adherence with virological target engagement, immunological target engagement, brain-pathway engagement, and brain and cognitive outcomes. State-matched follow-up combines event-triggered sampling for reactivation, serial reservoir monitoring for persistence, and acute-to-recovery tracking. Longitudinal cohorts, active-comparator studies, natural experiments, and randomized trials provide complementary approaches to evaluating intervention effects and their biological pathways.

## 5 Vaccination and cognitive outcomes

Vaccination can modify future infection, reactivation, or disease severity before downstream cognitive outcomes arise. Its actions include pathogen-specific protection and broader changes in immune responsiveness. Randomized zoster trials establish protection against herpes zoster, while dementia evidence comes primarily from observational and quasi-experimental studies (35, 36, 92). Together, these designs connect clinical findings with the immune mechanisms that prospective cognitive studies can examine.

### 5.1 Human evidence across vaccine settings

Zoster vaccination offers complementary population-level comparisons. Using age eligibility as an instrument for vaccine receipt, a Wales natural experiment estimated a 3.5-percentage-point reduction in new dementia diagnoses over seven years, corresponding to a 20.0% relative reduction (36). Canadian natural experiments reported lower dementia incidence associated with vaccine-program eligibility (93). The receipt-based and eligibility-based analyses motivate complementary prospective tests of vaccine responses and subsequent infection burden.

Active comparators distinguish vaccine strategies. Recombinant versus live zoster vaccination was associated with 17% more dementia-diagnosis-free time over the analyzed interval (35). A two-dose recombinant-vaccine cohort reported adjusted hazard ratios of 0.49 against unvaccinated and 0.73 against Tdap-vaccinated comparators (94). These contrasts evaluate vaccination across different clinical alternatives. **Table 5** summarizes each study’s population, exposure, and reported effect measure.

**Table 5:** Selected human studies of viral exposure and intervention.

Study and design	Sample and follow-up	Reported result	Exposure or transition
Eyting et al. (36) Wales natural experiment	282,541 adults; 7 years	Dementia risk reduction: 3.5 percentage points (0.6–7.1); 20.0% relative (6.5–33.4).	Vaccine receipt estimated from age eligibility
Pomirchy et al. (93) Canada natural experiments	232,124 Ontario patients; 5.5 years	Eligibility-associated reduction: 2.0 percentage points (0.4–3.5); replication threshold 2.0 (0.2–3.8).	Program eligibility at policy thresholds
Taquet et al. (35) Active-comparator cohort	103,837 matched pairs; median 4.15 years; analysis to 6 years	17% more dementia-diagnosis-free time; 164 additional days among those later diagnosed.	Recombinant versus live zoster vaccine
Rayens et al. (94) Matched cohort	65,800 vaccinated; 263,200 unvaccinated matches	Dementia aHR: 0.49 (0.46–0.51) versus unvaccinated; 0.73 (0.67–0.79) versus Tdap.	Two-dose recombinant zoster vaccination
Devanand et al. (95) VALAD randomized trial	120 HSV-seropositive adults with early symptomatic AD; 78 weeks	Greater ADAS-Cog worsening with valacyclovir: 3.93 points (1.03–6.83).	Valacyclovir versus placebo
Pawlowski et al. (12) Before-and-after cohort	52 completers with HCV clearance; 5–6 months	CVLT 50.9 → 54.1 (p = 0.0005); RFFT 77.2 → 86.1 (p < 0.0001).	Successful direct-acting antiviral treatment
Warren-Gash et al. (89) DIAN longitudinal study	Cognition n = 164; MRI n = 149; median 3.0 years	HSV-1 seropositivity: no association with faster cognitive decline or whole-brain atrophy.	Baseline HSV-1 serology
Sun et al. (96) Cohort meta-analysis	6 cohorts; 2,087,195 participants; 4–13 years	Dementia RR: 0.69 (0.57–0.83).	Influenza vaccination

Effect-estimate intervals are 95% confidence intervals. AD, Alzheimer disease; ADAS-Cog, Alzheimer’s Disease Assessment Scale–Cognitive Subscale; aHR, adjusted hazard ratio; CVLT, California Verbal Learning Test; DIAN, Dominantly Inherited Alzheimer Network; RFFT, Ruff Figural Fluency Test; RR, risk ratio; Tdap, tetanus–diphtheria–acellular pertussis vaccine.

Influenza and SARS-CoV-2 vaccination broaden the evidence beyond reactivation. Influenza-vaccine meta-analyses and cohorts report associations with lower dementia or neurodegenerative-disease incidence (96–99). A breakthrough-infection cohort found lower post-acute risk in vaccinated than unvaccinated infected individuals (100). These observational findings motivate cognition-specific follow-up, with post-acute recovery and incident dementia assessed on their respective timescales.

### 5.2 Pathogen-specific protection and adjuvant biology

Pathogen-specific protection provides a testable route from vaccination to lower infection-related brain stress. Fewer infections or reactivation episodes, and less severe illness, would reduce repeated inflammatory and vascular exposure. Zoster-vaccine trials establish clinical reactivation prevention, and human VZV antigen-challenge studies show changes in local cellular responses (20, 92). Prospectively linking reduced event burden to cognitive change would assess this pathway.

AS01 provides a defined mechanism for studying adjuvant effects. Its MPL and QS-21 components cooperate in an early innate response; preclinical work identified an IL-12/IL-18-associated IFN-gamma circuit involving NK and resident CD8 T cells that supported antigen-specific CD4 responses (101). Human lymph-node explants also show innate-cell activation by AS01 (102). These findings explain enhanced adaptive immunity and identify immune responses to measure alongside long-term cognitive outcomes.

Adjuvant-associated epidemiology extends this mechanistic question. A cohort comparing AS01-adjuvanted shingles and respiratory syncytial virus vaccination with other strategies reported lower dementia risk (103). Combined with the experimental evidence, this finding motivates assessment of antigen-specific responses, durable immune reprogramming, and subsequent infection burden. Measuring these processes alongside cognition would distinguish event prevention from broader immune effects.

### 5.3 Heterologous immune effects and mechanistic tests

Heterologous responses can be characterized through functional immune profiling. In 323 adults, multi-omics analysis of the bacterial BCG vaccine identified baseline epigenetic cell states associated with trained-immunity responses (21). A randomized MMR study demonstrated functional and metabolic reprogramming of gamma-delta T cells (104). These vaccine studies provide functional assays for investigating durable immune reprogramming in prospective cognition research.

A prospective vaccine study could link baseline immunity, post-vaccination responses, subsequent infection or reactivation, and repeated cognition. Natural experiments establish the population-level question, while antigen-challenge and vaccination studies provide feasible immune measurements (20, 21, 36). A nested mechanistic component could examine event prevention, improved specific control, and heterologous responsiveness alongside the primary clinical comparison. **Figure 2** integrates intervention exposure, biological responses, and cognitive outcomes.

## 6 Antiviral interventions and biological reversibility

Antivirals perturb an existing infection and provide a complementary test of reversibility. HIV suppression and HCV clearance represent different treatment-anchored transitions (12, 44). Anti-herpetic cohorts and meta-analysis also associate treatment with lower dementia incidence (105, 106). Relating treatment timing, virological response, and immune recovery to cognitive trajectories can identify the biological processes accompanying clinical change.

The VALAD randomized trial directly evaluated valacyclovir in early symptomatic Alzheimer disease. In 120 HSV-seropositive adults, 78 weeks of treatment produced greater ADAS-Cog worsening than placebo: a between-group difference of 3.93 points (95% CI, 1.03–6.83) (95). The trial provides a treatment-assignment estimate for this defined disease stage, complementing the suppression and clearance transitions examined in HIV and HCV.

Clinical effects and biological responses answer complementary questions. Randomization estimates the effect of treatment assignment, while virological and immune measurements determine which processes changed. CSF-escape and HCV-clearance studies characterize differences between systemic suppression, compartmental activity, and recovery (8, 12, 22). The stages in **Figure 2** integrate these biological measurements with brain and cognitive outcomes.

## 7 Translating the evidence into prospective and experimental studies

The three-state framework translates into different observation windows and experimental comparisons. Human shedding studies resolve brief local events, HIV cohorts identify compartmental discordance, and longitudinal COVID-19 studies connect early biology to later outcomes (8, 9, 13). **Figure 2** summarizes event-triggered, persistence-focused, and acute-to-recovery follow-up; **Table 2** links measurements to timing and purpose. The illustrative designs below can be adapted to the virus, clinical transition, and expected event frequency.

### 7.1 Reactivation-anchored longitudinal studies

Reactivation studies can test whether impaired control precedes an episode and whether a host or cognitive response follows it. Dense HSV-2 sampling shows that activity changes faster than routine cohort visits (9, 10). A feasible design combines a stable baseline, home sampling from an appropriate accessible site around symptoms or detected shedding, and early-recovery blood and cognitive assessments. Events are defined virologically or clinically, with within-person event-free intervals providing the primary comparison.

Measurements before and after an event distinguish alternative sequences. Local functional changes preceding shedding would implicate reduced surveillance; inflammation preceding reactivation would favor a host-triggered or feedback process. VZV antigen-challenge and activation studies provide precedents for focused cellular assays (11, 20). Estimating the timing and magnitude of these responses links local containment to the subsequent cognitive trajectory.

### 7.2 Recovery during viral suppression or after clearance

Persistence studies can anchor follow-up to treatment initiation or documented clearance. HIV reservoir and CSF-escape findings motivate serial measurements beyond plasma viral load, while HCV treatment offers a defined clearance transition (8, 12, 23). An illustrative cohort could assess participants before treatment, near the first virological response, and at approximately three, six, and twelve months, with sampling integrated into clinical care.

The analysis compares viral reduction, immune normalization, and cognitive recovery. HIV studies can focus on residual reservoirs and myeloid or metabolic pathways; HCV studies can incorporate liver disease and hepatic recovery. Kynurenine and ART-suppressed SIV findings nominate candidate host processes (47, 48). Concordant host and cognitive improvement supports a reversible component, whereas persistent impairment after host normalization directs attention to prior injury, disease stage, treatment effects, or comorbidity.

### 7.3 Acute-to-recovery studies of post-infectious cognition

Acute-to-recovery studies can distinguish continuing antigen exposure from delayed host resolution. Early multi-omics, longitudinal complement profiling, and pre-infection imaging provide empirical precedents (13, 28, 51). Illustrative visits during acute illness, early convalescence, and at three, six, and twelve months could pair antigen measurements with a focused host pathway and standardized cognition. Recording reinfections and treatment changes preserves the interpretation of recovery trajectories.

Comparisons between participants with persistent symptoms, recovered infected participants, and a suitable background group address complementary questions. Within the infected cohort, antigen and pathway trajectories can be related to persistent impairment versus recovery. CSF profiling or barrier imaging can be nested in appropriate subsets, following existing post-COVID studies (14, 25). Paired measurements can relate central immune and vascular responses to antigen dynamics and cognitive recovery.

### 7.4 Experimental perturbation of a defined mechanistic link

Experiments can test the mechanism suggested by a human trajectory. Neuron–immune systems resolve noncytolytic HSV control, lung–brain systems localize barrier injury, and post-encephalitic models permit complement perturbation (16, 64, 67). Comparing antiviral suppression, host-pathway inhibition, and their combination while measuring viral activity and the same downstream phenotype can distinguish replication-dependent injury, a persistent host program, and contributions from both.

Perturbation timing separates prevention from maintenance and reversibility. Intervention before infection tests prevention; treatment after documented viral control tests an established host response. Inflammatory-memory and neurodegeneration models show how immune responses can remain altered after the initiating challenge (70, 74). Concurrent viral-load and viability measurements assess antiviral control, cellular health, and neural recovery together.

### 7.5 Feasibility, outcome alignment, and analysis

A core cohort with nested mechanistic substudies offers a practical balance of scale and depth. Biobanks link infection histories with dementia, plasma proteins, and imaging phenotypes (78, 107, 108). Core visits could retain event verification, a focused blood panel, and repeated cognition; subsets could add dense sampling, functional assays, CSF, or imaging. Pilot estimates of event yield, within-person variability, and retention would guide sampling intensity and sample-size simulations.

Analysis follows the clinical or biological contrast. Target-trial emulation defines eligibility, assignment, time zero, follow-up, and outcome in observational intervention studies (109). For longitudinal cohorts, within-person mixed-effects and lagged analyses can relate changes around events to later cognition, with time-varying factors assigned their role in a question-specific causal model. This approach complements the longitudinal-cohort, active-comparator, natural-experiment, and randomized-trial designs summarized in **Figure 2**.

Outcome-specific analysis also accounts for participation, mortality, and repeated testing. Selection can create collider bias, death can preclude dementia diagnosis, and practice effects or dropout can alter cognitive trajectories (27, 62, 63, 110). Appropriate comparison groups, missing-data handling, and competing-risk analyses address these issues. Sensitivity analyses and genetic approaches offer complementary evidence under their respective assumptions (111, 112).

## 8 Discussion and research priorities

Organizing evidence by infection state identifies when a biological process can be observed or modified. Brief HSV reactivation, compartmental HIV activity during systemic suppression, and prolonged responses after respiratory infection present different opportunities (8, 9, 52). The three prototypes therefore function as guides to timing and mechanism, linking exposure history to the process active when cognitive change occurs.

The first priority is to resolve the sequence between impaired control, viral activity, and host response. Local containment and noncytolytic control studies identify protective processes, while neuronal stress can trigger reactivation (10, 16, 42). Event-anchored observation can distinguish reduced surveillance from host-triggered activity and subsequent inflammatory effects. These alternatives determine which transition an intervention should target.

A second priority is to connect accessible immune measurements with brain-relevant compartments. HIV CSF-escape and post-COVID CSF or barrier studies identify distinct systemic and central patterns (14, 22, 25). Nested paired measurements can establish which peripheral markers track local or central processes, enabling informative follow-up at cohort scale.

A third priority is to distinguish continuing antigen exposure from persistent host programming. Post-acute antigen detection, longitudinal complement abnormalities, and experimental immune memory nominate different maintenance processes (4, 28, 74). Their trajectories during recovery or treatment can identify a candidate link for antiviral or host-pathway perturbation. This connects human temporal evidence with a focused experimental test.

The mechanistic evidence supports complementary next steps. Human imaging and tissue models support neurovascular mechanisms, whereas complement-dependent synaptic loss has strong perturbational evidence in selected experimental systems (25, 64, 67, 79). Kynurenine associations provide measurable metabolic candidates across infections (47, 69). **Table 4** connects these findings with focused targets for longitudinal observation, compartmental measurement, and pathway perturbation.

Vaccination and antiviral studies highlight modifiability and treatment context. Zoster natural experiments and active-comparator cohorts associate vaccination with more favorable dementia outcomes, while VALAD found greater cognitive worsening with valacyclovir in early symptomatic Alzheimer disease (35, 36, 93, 95). Prospective studies can combine a defined clinical comparison with focused viral and immune measurements to test how infection state and disease stage shape the response to intervention.

Matching cognitive endpoints to biological transitions makes reversibility and long-term progression separately testable. Persistent symptoms, multi-year trajectories, and dementia diagnoses capture different clinical processes and timescales (6, 36, 57). Disease-stage biomarkers, repeated-test analysis, and competing-risk methods support outcome-specific evaluation (27, 60, 62). This research agenda links a defined infection process to its immune consequences and tests whether modifying that process improves the relevant cognitive outcome.
